# SSTM-IS: simplified STM method based on instance selection for real-time EEG emotion recognition

**DOI:** 10.3389/fnhum.2023.1132254

**Published:** 2023-06-01

**Authors:** Shuang Ran, Wei Zhong, Danting Duan, Long Ye, Qin Zhang

**Affiliations:** ^1^Key Laboratory of Media Audio & Video, Ministry of Education, Communication University of China, Beijing, China; ^2^State Key Laboratory of Media Convergence and Communication, Communication University of China, Beijing, China

**Keywords:** brain-computer interface, EEG signals, real-time emotion recognition, instance selection, transfer learning

## Abstract

**Introduction:**

EEG signals can non-invasively monitor the brain activities and have been widely used in brain-computer interfaces (BCI). One of the research areas is to recognize emotions objectively through EEG. In fact, the emotion of people changes over time, however, most of the existing affective BCIs process data and recognize emotions offline, and thus cannot be applied to real-time emotion recognition.

**Methods:**

In order to solve this problem, we introduce the instance selection strategy into transfer learning and propose a simplified style transfer mapping algorithm. In the proposed method, the informative instances are firstly selected from the source domain data, and then the update strategy of hyperparameters is also simplified for style transfer mapping, making the model training more quickly and accurately for a new subject.

**Results:**

To verify the effectiveness of our algorithm, we carry out the experiments on SEED, SEED-IV and the offline dataset collected by ourselves, and achieve the recognition accuracies up to 86.78%, 82.55% and 77.68% in computing time of 7s, 4s and 10s, respectively. Furthermore, we also develop a real-time emotion recognition system which integrates the modules of EEG signal acquisition, data processing, emotion recognition and result visualization.

**Discussion:**

Both the results of offline and online experiments show that the proposed algorithm can accurately recognize emotions in a short time, meeting the needs of real-time emotion recognition applications.

## 1. Introduction

Brain-Computer Interface (BCI) is a communication system that does not depend on the output path composed of peripheral nerves and muscles (Wan et al., 2005; Schalk et al., 2008; Hamadicharef, 2010). The BCI allows users to control computers or other devices through brain activity and involves many research fields such as medicine, neurology, signal processing, and pattern recognition. In recent years, the affective BCI (aBCI) has attracted great interests which endows BCI system with the ability to detect, process and respond to the affective states of humans using EEG signals (Accordino et al., 2007; Mu and Lu, 2020; Wu et al., 2022b). The aBCI has shown great development potential in many application fields, for example, it can help the patients with psychological diseases establish effective social interaction (Murias et al., 2007; Lee et al., 2014); remind the driver to better focus on the driving to avoid traffic (Gao et al., 2019); make the machine analyze the emotion of human and provide emotional companionship (Park et al., 2009).

Emotion is one of the most important physiological and psychological states of the human body. Modern medicine believes that emotion is directly related to human health (Luneski et al., 2010). Prolonged negative emotion can reduce creativity and cause a loss of focus, even cause anxiety and depression (Walsh et al., 2005). Accurate and reliable emotion monitoring can, on one hand, help to restore emotion and enhance concentration and, on the other hand, help service providers to analyze user preferences and thus provide more personalized products and services (Mauss and Robinson, 2009). Currently, emotion monitoring is based on two main types of physiological signals: subjective physiological signals such as voice and expression, and objective physiological signals such as EEG and ECG. Since the EEG signal has the advantages of high temporal resolution, not easy to pretend and the popularity of non-invasive portable acquisition devices, it has been widely used for emotion detection.

**Figure 1:**
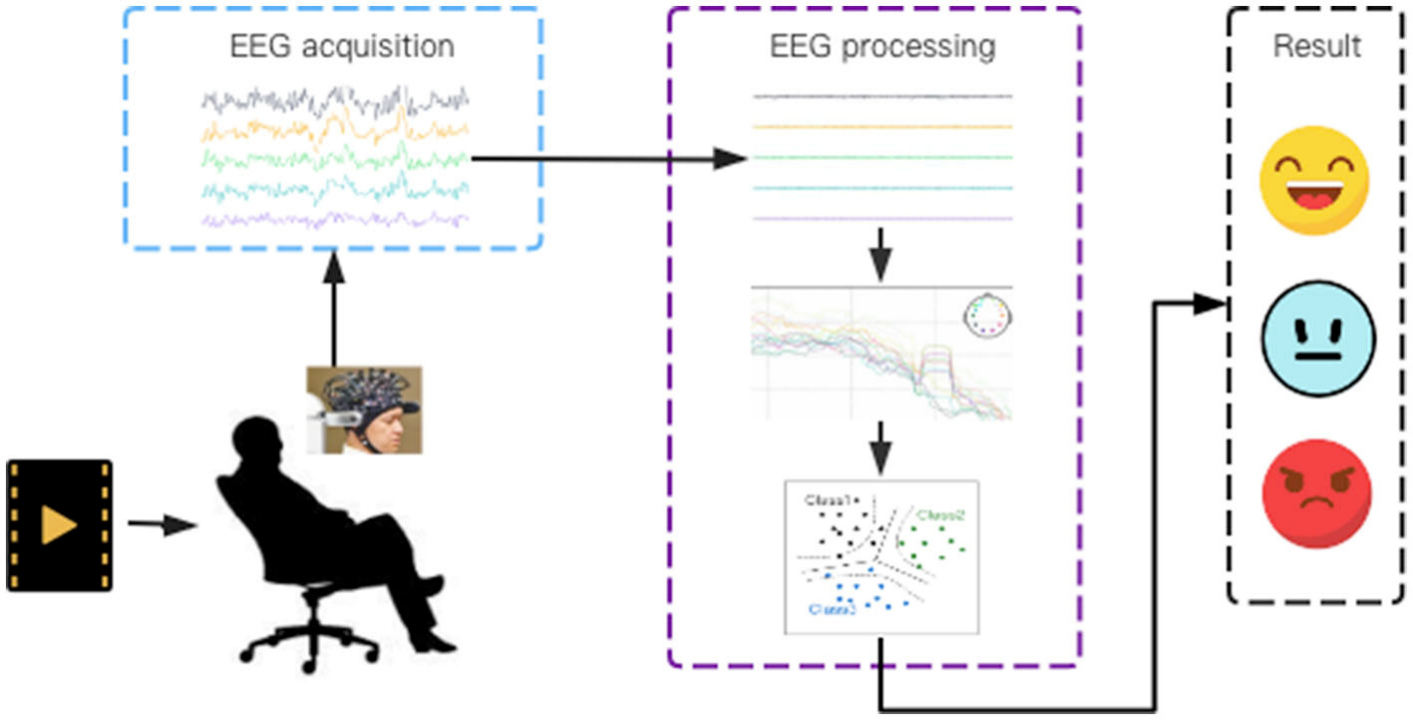
The workflow of EEG signal-based emotion recognition: stimulating the subject's emotions, collecting EEG signals and recognizing emotions based on the features extracted from the pre-processed EEG signals.

As shown in Figure 1, the workflow of EEG signal-based emotion recognition includes the acquisition of EEG signals, signal pre-processing, feature extraction and classification, and the output of recognition result. For the EEG processing, the acquired EEG signals need to be pre-processed first to improve the signal-to-noise ratio by the methods such as filtering, artifact removal and principal component analysis, because their amplitudes are very small and susceptible to be interfered by other electrophysiological signals. And then, the features in time domain, frequency domain and time-frequency domain are extracted and decoded to recognize the emotion of experimenter by using the recognition methods such as machine learning.

The existing methods of emotion recognition based on EEG signals have the following two limitations:

- Most of the methods use the models trained on existing data to test the new subject for real-time emotion recognition. However, the EEG signals are non-stationary and the training and testing samples must be collected from the same individual or even the same test environment, otherwise the recognition accuracy will drop dramatically. This means building universal models that work across subjects is challenging.
- Most of the existing cross-subject methods need to analyze the results manually or process data offline, which require all the EEG data to be saved first. While the human emotions always change in real-time, the above methods are time-consuming and user-unfriendly, difficult to support the practical applications of real-time emotion recognition.

To address both of these points, our work aims to explore an algorithm that can recognize emotion in cross-subject situation quickly and accurately. By selecting the informative instances and simplifying the update strategy of hyperparameters, we propose a simplified style transfer mapping method based on instance selection (SSTM-IS), which can use a small amount of labeled data in the target domain to make the model adapt for the new subject in a short time. To verify the effectiveness of the proposed method, we compare and analyze the performance of representative methods in terms of accuracy and computing time on SEED (Zheng and Lu, 2015), SEED-IV (Zheng et al., 2019), and collected dataset by ourselves for emotion recognition. The experimental results show that our algorithm achieves the accuracy rate of 86.78% in the computing time of 7 s on the SEED dataset, 82.55% in 4 s on SEED-IV and 77.68% in 10 s on self-collected dataset. In addition, the work in this paper also includes the design and implementation of a real-time aBCI system to test the proposed algorithm in practical application. There are three key contributions in this work:

- By selecting the informative instances and simplifying the update strategy of hyperparameters in style transfer mapping, we propose the SSTM-IS algorithm to perform the cross-subject emotion recognition more accurately and quickly.
- We validate the proposed algorithm on both of the public and self-collected datasets. The experimental results demonstrate that the proposed algorithm can achieve higher accuracy in a shorter computing time, satisfying the needs of real-time emotion recognition applications.
- We design and implement a real-time emotion recognition system that integrates the modules of EEG signal acquisition, data processing, emotion recognition and result visualization. The applicability of the proposed algorithm is also verified online in a real case.

This paper is structured as follows: Section 2 briefly discusses the related works. The proposed emotion recognition algorithm is described in detail in Section 3. Subsequently in Section 4, the real-time emotion recognition system is developed and realized. And then Section 5 gives and analyzes both of the offline and online experimental results. Finally, some conclusions and future works are presented in Section 6.

## 2. Related works

As we know, the EEG signals are non-stationary and various people to people. The emotion recognition model trained on the existing dataset is often not applicable to new subjects. While the applications of real-time emotion recognition require algorithms to accurately and quickly recognize the EEG signals of a newcomer. A common solution is to introduce transfer learning into EEG emotion recognition (Jayaram et al., 2016; Wu et al., 2022a), so as to make the model adapt to new individuals. The core of transfer learning is to find the similarity between existing knowledge and new one, so can use the existing to learn the new. In transfer learning, the existing knowledge is denoted as the source domain, and the new knowledge to be learned is defined as the target domain. The distribution between the source domain and target domain are different but related to each other. It is necessary to reduce the distribution difference between the source and target domains for knowledge transfer.

For the EEG emotion recognition, one method is to use all unlabeled data from target domain to train the model, and enhance the model performance by reducing the differences between the source domain and target domain. Li et al. (2021) proposed the BiDANN-S framework for cross-subject emotion recognition, which reduces the differences across domains by adversarially training domain discriminators. Li et al. (2020b) used the adversarial training to adjust the marginal distribution of the shallow layers for reducing the difference between the source and target domains, while using correlation enhancement to adjust the conditional distribution of the deep layers for enhancing network generalization. Chen et al. (2021) proposed the MS-MDA network based on domain-invariant and domain-specific features, so that different domain data share the same underlying features, while preserving the domain-specific features. This kind of methods given above belong to deep learning algorithms, as we know, the deep learning algorithms use the back propagation for parameter optimization, and needs a large amount of data in model calibration phase to improve the recognition performance of new subjects. As a result, when a new subject comes, the consuming time for model calibration is relatively long and thus these algorithms are not suitable for the applications of real-time EEG emotion recognition. Another method mainly obtains the domain invariant features from the differences of multiple source domains to train a general model. In the training phase, it is not necessary to obtain any data from the target domain. Ma et al. (2019) proposed a new adversarial domain generalization framework, DResNet, in which the domain information was used to learn unbiased weights across subjects and biased weights specific to subjects. However, the performance of the model obtained by this method is generally worse than that of models with target domain data participating in the training. For example, the accuracy rate of the PPDA model with all target domain data participating in training is 1.30% higher than that of the PPDA_NC without target domain data involved in training (Zhao et al., 2021).

From the above analysis, it can be inferred that one of the ideas to obtain a real-time emotion recognition model with good performance is to integrate the two kinds of methods, that is, using a small amount of target domain data for supervised learning, and training a generalized model. Chai et al. (2017) proposed an ASFM to integrate both the marginal and conditional distributions within a unified framework, which achieves an accuracy of 83.51% on the SEED dataset. It should be noticed that on the selection of source domain, the works described above use all the subjects' source domain data to train the model without selection. However, the studies in Yi and Doretto (2010) and Lin and Jung (2017) have shown that the inappropriate selection of source domain data may cause the negative transfer. In addition, the large amount of data involved in model training phase increases the computing time and is not applicable to real-time emotion recognition. Li et al. (2020a) considered each subject as a source domain for multi-source domain selection and proposed a MS-STM algorithm which reduces the domain difference when using a small amount of labeled target data. Later, Chen et al. (2022) proposed a conceptual online framework FOIT in which they selected instance data from source domains, but the resulting recognition accuracy is not satisfactory and the framework is not verified in an actual scene.

On the real-time emotion recognition system, to the best of our knowledge, there are few reports on relevant work. Jonathan et al. (2016) developed a mobile application for processing and analyzing EEG signals, which can display the EEG spectrum, classify EEG signals, visualize, and analyze the classification results. However, this system does not realize the data upload function, and only uses the offline data that has been stored on the server. Nandi et al. (2021) proposed a real-time emotion classification system based on logistic regression classifier, and carried out the experiments of simulating real-time emotion recognition on DEAP. Weiss et al. (2022) compared several classifiers and chose the logistic regression to realize a real-time EEG emotion recognition system on SEED-IV. Although the above works put forward the concept of real-time emotion recognition system, they use the models trained on existing data to test the new subject, without model calibrating. Besides they all use the offline datasets or simulated real-time emotion recognition for verification, not performing the real-time emotion recognition applications in real scene.

According to the above analysis, the human emotions always change over time, and thus it is necessary to realize a real-time emotion recognition system with good performance in practical application. The existing transfer learning methods for EEG emotion recognition either use all the subjects' source domain data to train the model without selection, or consider each subject as a source domain for multi-source domain selection, which may cause negative transfer and increase the computing time. To address this problem, this paper proposes a simplified STM algorithm by optimizing the updating strategy of hyperparameters in STM and using SVM classifier with a one-vs-one scheme for parameter optimization. On the other hand, we also refine the granularity of selecting source domain data, and obtain the most informative instances to further enhance the generalization of the model. With the above improvements, the algorithm proposed can achieve higher accuracy by using a small amount of data for model calibrating in a short computing time, satisfying the need of real-time EEG emotion recognition applications.

## 3. Methods

**Figure 2:**
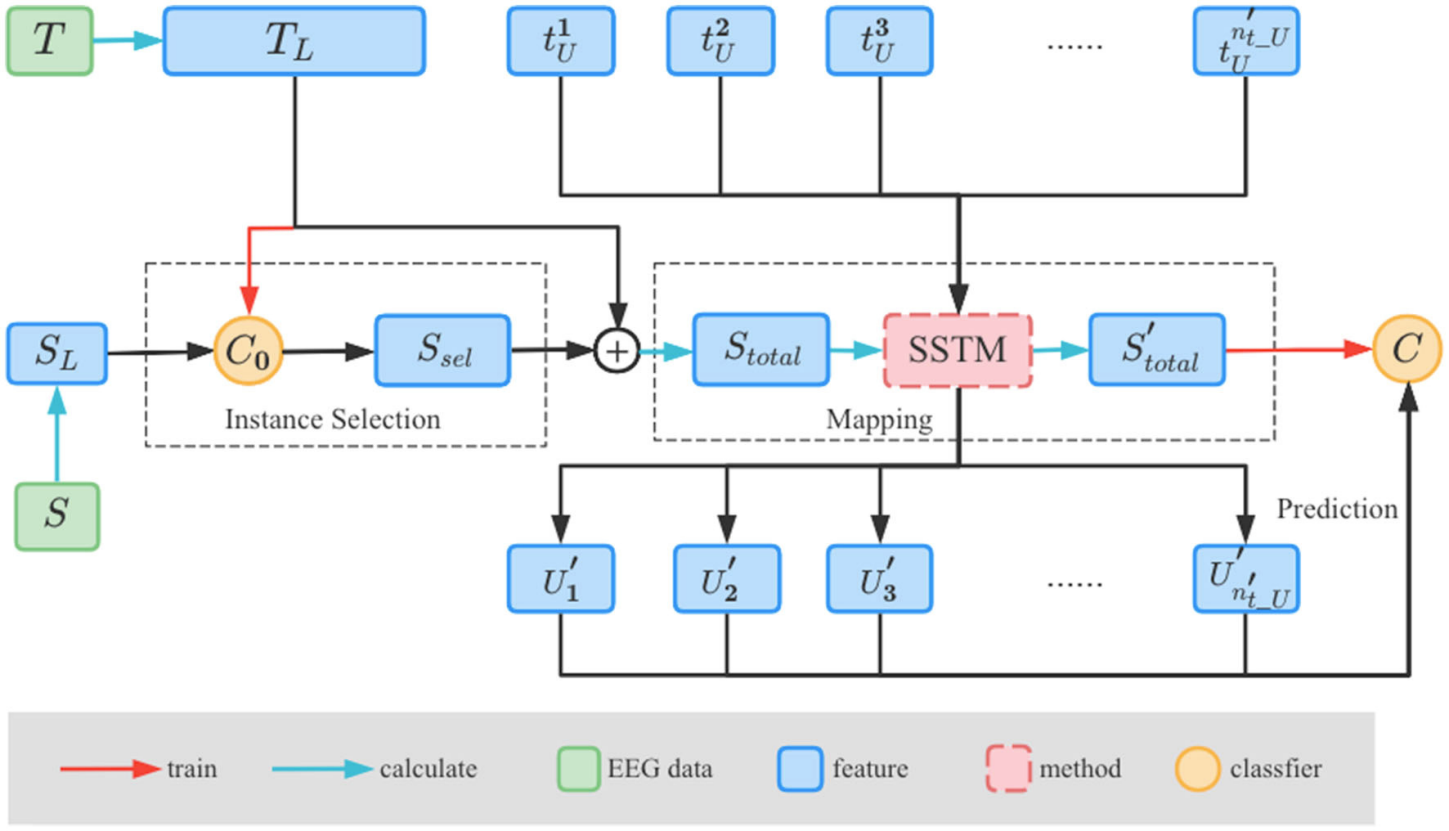
Framework of the proposed SSTM-IS algorithm.

In this section, we present the proposed SSTM-IS method which consists of two main steps as shown in Figure 2. Firstly, our work is to refine the granularity of selecting source domain data from different subjects to different sample instances, and select the most informative instances from the source domain through a classifier trained by the labeled data from the target domain to improve the recognition accuracy of new subject. And then by simplifying the updating strategy of hyperparameters in STM, a simplified STM (SSTM) algorithm is developed to make the distributions of source and target domains more similar.

Define the source domain as *S* = {*s*_*i*_, *i* = 1, 2, ..., *n*_*s*_} and its corresponding label is $L=\left\{y_{s}^{1},y_{s}^{2},...,y_{s}^{n_{s}}\right\}$. The target domain is *T* = {*t*_*i*_, *i* = 1, 2, ..., *n*_*t*_} which is divided into the labeled and unlabeled parts, and the labeled part corresponds to the label *L*_*T*_. Here, *n*_*s*_ and *n*_*t*_ are, respectively, the sample numbers of source domain and target domain data. *S* and *T* have different marginal distributions. The DE features (Duan et al., 2013; Shi et al., 2013) of the source domain are extracted and denoted as $S_{L}=\left\{s_{L}^{i}\in R^{m}|i=1,2,...,n_{s}^{{}^{\prime }}\right\}$, where $n_{s}^{{}^{\prime }}$ represents the number of DE feature samples of source domain and *m* represents the feature dimension. Similarly for the target domain, the DE features of the labeled and unlabeled data are $T_{L}=\left\{t_{L}^{i}\in R^{m}|i=1,2,...,n_{t\text{\_}L}^{{}^{\prime }}\right\}$ and $T_{U}=\left\{t_{U}^{i}\in R^{m}|i=1,2,...,n_{t\text{\_}U}^{{}^{\prime }}\right\}$, respectively, where $n_{t\text{\_}L}^{{}^{\prime }}$ and $n_{t\text{\_}U}^{{}^{\prime }}$ represent the feature numbers of the labeled and unlabeled target domain data. $U^{{}^{\prime }}=\left\{U_{i}^{{}^{\prime }}\in R^{m}|i=1,2,...,n_{t\text{\_}U}^{{}^{\prime }}\right\}$ represents the features mapped from *T*_*U*_.

### 3.1. Instance selection

Using the source domain data and target domain data for transfer learning can build a robust emotion recognition model. Among the existing EEG emotion recognition algorithms, some methods use all of the subjects' data to train the model and do not perform the selection of source domain data. The others consider each subject as a source domain for multi-source domain selection, and the selected source domain data are all involved in the model training. However, the inappropriate selection of source domain data may cause the negative transfer. In addition, the large amount of data involved in model training increases the computing time and is not applicable to real-time recognition. Inspired by the idea of sample query in active learning (Hossain et al., 2018), we propose to refine the granularity of selecting source domain data from different subjects to different sample instances. The specific strategy is training a classifier with labeled target domain data and using the classifier to select the most informative instances from the source domain to improve the emotion recognition accuracy of new subject.

For the procedure of instance selection shown in Figure 2, we first train an SVM classifier *C*_0_ by using the DE feature *T*_*L*_ of the labeled target domain data and its corresponding label *Y*_*L*_. Here, *C*_0_ is the same for all the domain subjects, and is trained by the sessions with different emotion categories from labeled target domain. The probability of each sample in source domain denoted as *w*_*i*_ is then predicted by *C*_0_ and taken as the information contained in each sample instance:


(1) $$\begin{array}{l} w_{i}=Acc\left(C_{0},s_{L}^{i}\right),\;i=1,2,...,n_{s}^{{}^{\prime }}. \end{array}$$

Subsequently, the instances can be selected according to their prediction probabilities *w*_*i*_. To avoid the unbalanced distribution, we sort the instances of the source domain within each emotion category according to the amount of information it contains as *S*_*L*_*sort*_, and select the top *k* samples of instance data with the highest probabilities for each emotion category as the top *k* highest informative instances *S*_*sel*_:


(2) $$\begin{array}{l} S_{L\text{\_}sort}=\left\{S_{L}^{i}\;sorted\;by\;w_{i}\;and\;grouped\;by\;y_{s}^{i}\right\}=S_{L}^{1}\cup S_{L}^{2}\cup ...\cup S_{L}^{n_{c}}, \end{array}$$

(3) $$\begin{array}{l} S_{sel}=S_{L}^{1}\left[1:k\right]\cup S_{L}^{2}\left[1:k\right]\cup ...\cup S_{L}^{n_{c}}\left[1:k\right], \end{array}$$

where $y_{s}^{i}$ is the emotion label of each instance in source domain, and *n*_*c*_ is the number of emotion categories. And thus, we can get the informative instance data *S*_*sel*_ for the subsequent transfer learning to reduce the data redundancy and improve the computing efficiency.

### 3.2. Simplified STM

The STM algorithm (Li et al., 2020a) solves a style transfer mapping to project the target domain data *T* to another space, where the differences between the target domain *T* and the source domain *S* are reduced. In this way, the classifier *C* trained by the source domain data can be used for the classification of the target domain data in a specific space. As shown in Figure 2, the category number for classifiers of *C*_0_ and *C* is consistent with those of emotions recognized. Assuming the DE features of the source and target domains obey Gaussian distribution, the labeled target domain data *T*_*L*_ can be mapped by the Gaussian model into:


(4) $$\begin{array}{l} O=\left\{o^{i}\in R^{m}\right\},\;o^{i}=\mu_{c}+min\left\{1,\frac{\rho }{d\left(t_{L_{c}}^{i},c\right)}\right\},\;i=1,...,n_{t\text{\_}L}^{{}^{\prime }}, \end{array}$$

(5) $$d(t_{L_{c}}^{i},c)=\sqrt{{\left(t_{L_{c}}^{i}-\mu_{c}\right)}^{T}\sum_{c}^{-1}(t_{L_{c}}^{i}-\mu_{c})},$$

where *o*^*i*^ is the mapped value of $t_{L}^{i}$ by the Gaussian model, $d\left(t_{L_{c}}^{i},c\right)$ is the Mahalanobis distance in emotion category of *c* (*c* = 1, 2, ..., *n*_*c*_), $t_{L_{c}}^{i}$ is the target domain data corresponding to *c*, and μ_*c*_ is the average of the instances labeled with *c* in *S*_*sel*_. Here, ρ is used to control the deviation between *o*^*i*^ and μ_*c*_.

Suppose the affine transformation from *o*^*i*^ back to $t_{L}^{i}$ is represented as *Ao*^*i*^+*b*, the parameters *A*∈*R*^*m*×*m*^ and *b*∈*R*^*m*^ can be learned by optimizing the weighted square error with regular terms:


(6) $$\underset{A\in R^{m\times m},b\in R^{m}}{min}\;\sum_{i=1}^{{n^{\prime }}_{t\_L}}{\left\|Ao^{i}+b-t_{L}^{i}\right\|}_{2}^{2}+\beta {\left\|A-I_{m\times m}\right\|}_{F}^{2}+\gamma {\left\|b\right\|}_{2}^{2},$$

where the hyperparameters β and γ are used to control the state between non-transfer and over-transfer. The STM method (Li et al., 2020a) updates the values of β and γ in each iteration of calculation, spending much computing time. Through the experiment, it is found that the fixed values of β and γ can be obtained to shorten the computing time without reducing the accuracy. So we select β and γ as the fixed constants, and then Equation (6) can be solved as follows:


(7) $$\begin{array}{l} A=QP^{-1},\;b=\frac{1}{\hat{f}}\left(\hat{t}-Aô\right), \end{array}$$

(8) $$Q=\;\sum_{i=1}^{{n^{\prime }}_{t\_L}}f_{i}t_{L}^{i}{\left(o^{i}\right)}^{T}-\frac{1}{\hat{f}}\hat{t}{\hat{o}}^{T}+\beta I_{m\times m},$$

(9) $$P=\;\sum_{i=1}^{{n^{\prime }}_{t\_L}}f_{i}o^{i}{\left(o^{i}\right)}^{T}-\frac{1}{\hat{f}}\hat{o}{\hat{o}}^{T}+\beta I_{m\times m},$$

(10) $$\hat{o}=\;\sum_{i=1}^{{n^{\prime }}_{t\_L}}f_{i}o^{i},$$

(11) $$\hat{t}=\;\sum_{i=1}^{{n^{\prime }}_{t\_L}}f_{i}t_{L}^{i},$$

(12) $$\hat{f}=\;\sum_{i=1}^{{n^{\prime }}_{t\_L}}f_{i}\gamma ,$$

where the parameter *f*_*i*_ is the confidence of $t_{L}^{i}$ to *o*^*i*^. Since the parameter γ is fixed, $\hat{f}$ will not change with iteration, which reduces the computing time.

The specific pseudo code of SSTM-IS algorithm is shown in Algorithm 1. According to the description given above, we first select the most informative instance samples from the source domain, and then perform the transfer learning between the selected instances and the labeled target domain data by simplifying the updating strategy of hyperparameters in STM, improving the emotion recognition accuracy and increasing the computing speed.

**Algorithm 1:**
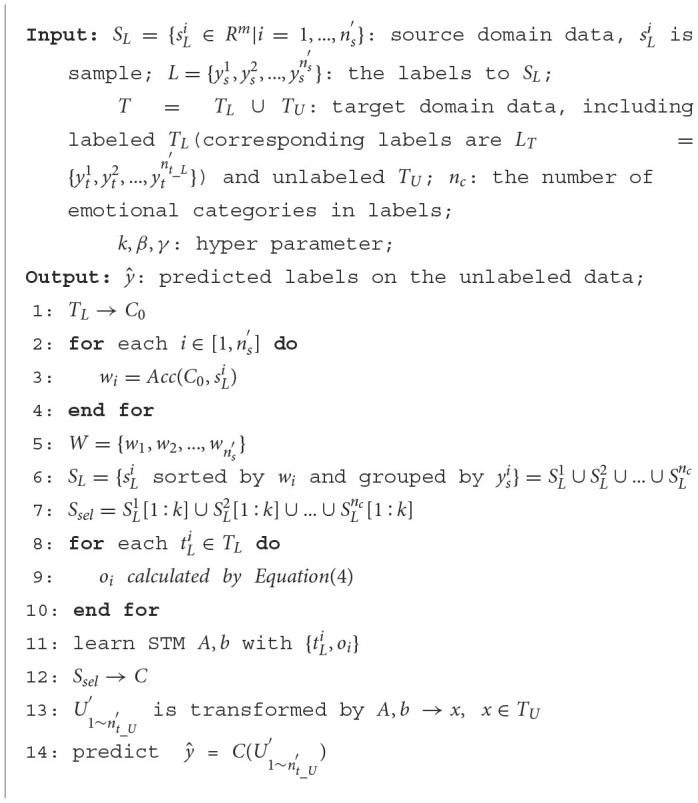
Simplified STM based on instance selection.

## 4. Real-time emotion recognition system

**Figure 3:**
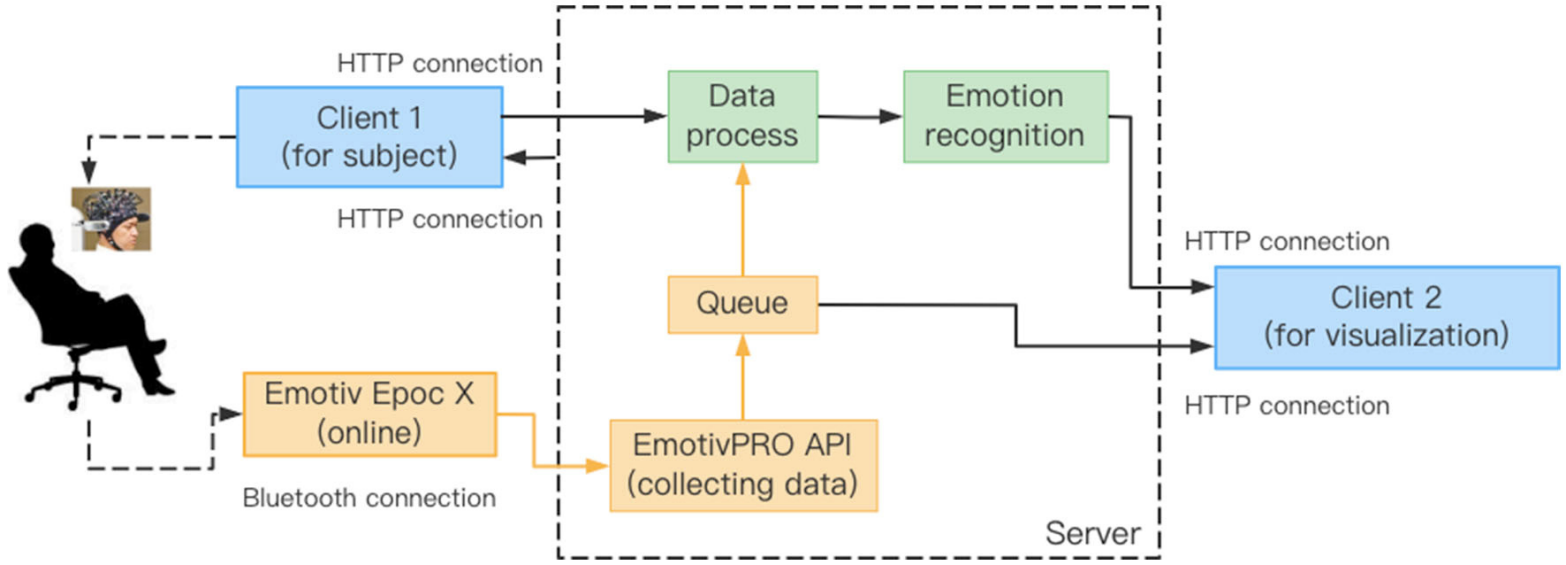
The flowchart of online emotion recognition.

Most of the existing emotion recognition systems based on EEG signals are limited to manual offline for data processing and result analysis. This way of offline processing needs to load all the data first, and thus cannot perform the real-time emotion recognition obviously. The SSTM-IS algorithm proposed in this paper can establish a model suitable for new subjects from existing data in a short computing time, and can be well-applied to real-time emotion recognition. To verify the effectiveness of the SSTM-IS algorithm in practical use, we simulate the real-time emotion recognition in on-line situation. The system framework is shown in Figure 3.

**Figure 4:**
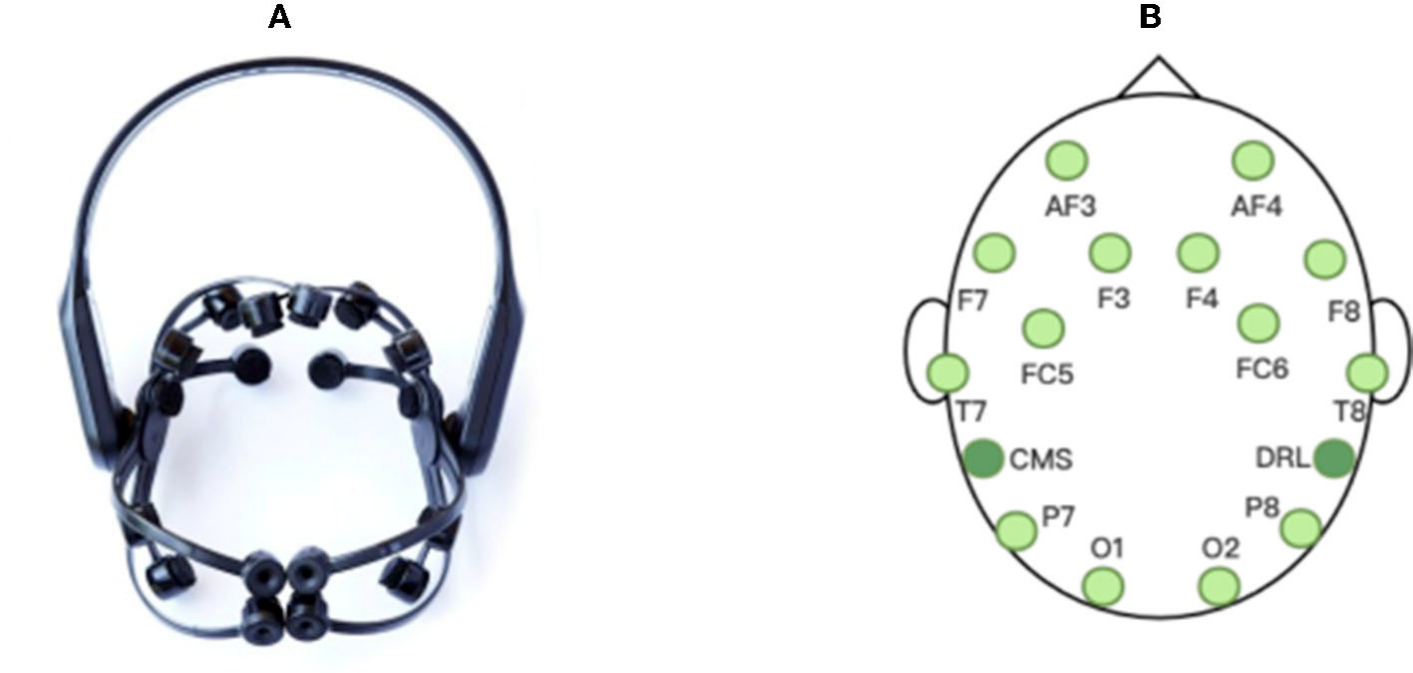
The EEG acquisition device used in our experiments: **(A)** Emotiv EPOC X, **(B)** the electrode distribution.

For the actual online situation, we develop a real-time EEG emotion recognition system that integrates EEG signal acquisition, processing, emotion recognition and result visualization, as shown in Figure 3. The system uses an Emotiv EPOC X with 14-channel electrodes to collect EEG signals, namely AF3, F7, F3, FC5, T7, P7, O1, O2, P8, T8, FC6, F4, F8, AF4 with additional two reference electrodes CMS and DRL, as shown in Figure 4. The sampling frequency of Emotiv EPOC X is 256 Hz and the bandwidth is 0.20–43 Hz. The Emotiv EPOC X is connected to the server by Bluetooth, and the EEG signals of the subjects are recorded by calling the EmotivPRO API. Here, the Client 1 is the experimental interface of the subject, which is used to realize the interaction between the subject and the server. In the experiment, the necessary prompts can be given to subjects by Client 1, allowing them to control the experimental process according to their own experiences, such as the length of rest time. We collected and stored the EEG data of the subjects who have experimented on this system before as the source domain data. As shown in Figure 3, when a new subject comes, he receives video stimulation, conducts subjective evaluation, and controls the experimental process through the interface of Client 1. The server follows the experimental paradigm to control the experimental process, stores and processes experimental data, as well as responds to the requests from pages. In the work process, the server collects the first three sessions of EEG data from the new subject as the labeled target domain data, and uses these labeled data to train a classifier for selecting the informative instance data from source domain. And then the simplified STM is called to obtain an emotion recognition model by using these labeled data and selected instance data. Subsequently, the server pre-processes the raw EEG data within every segment of 10 s by using the MNE-Python library for bandpass filtering of 0.10–50 Hz and notching filter for denoising. And then the DE features are extracted to put into the trained model for real-time emotion recognition. Once the model training is completed, the system only needs several milliseconds to output a emotion prediction for a 10s data segment. Finally, the server feeds the raw EEG signals and recognition results into the Client 2 for visualization. Here, the Client 2 is used to monitor the state of the subject, present video stimuli, visualize the raw EEG signals as well as their spectral maps and topographic maps, and analyze the real-time emotion recognition results.

## 5. Experiments and results

### 5.1. Datasets and settings

In order to verify the performance of the proposed SSTM-IS algorithm, we select two public datasets SEED (Zheng and Lu, 2015) and SEED-IV (Zheng et al., 2019), as well as the collected 14-channel EEG signal dataset for offline and online experiments. The SEED dataset (Zheng and Lu, 2015) selected 15 Chinese film clips with the types of positive, neutral, and negative as visual stimuli, and each clip is about 4 min. The 15 subjects conducted three experiments by using the same stimuli with an interval of 1 week, and 3,394 samples had been collected for each subject in one experiment. The SEED-IV dataset (Zheng et al., 2019) selected 24 movie clips with four emotions of happiness, sadness, fear, and neutral (6 clips for each emotion), and the duration of each movie clip is about 2 min. The 15 subjects conducted three groups of experiments which used completely different stimuli at different times, and 822 samples had been collected in one experiment. Both SEED and SEED-IV datasets use 62-channel acquisition equipment to record EEG signals of subjects at a sampling rate of 1,000 Hz, and pre-process the collected data as follows: downsampling to 200 Hz, using 0.3–50 Hz band-pass filter and extracting DE features. Both of the two datasets contain raw data and extracted features, and are available at http://bcmi.sjtu.edu.cn/~seed/index.html.

The dataset collected by ourselves recorded the EEG signals of 10 subjects at a sampling rate of 256 Hz with the Emotiv EPOC X, a 14-channel wireless portable EEG acquisition instrument. In the selection of stimuli videos, by taking into account the native language environment of the subjects, we selected 12 Chinese short videos with three emotions of positive, neutral, and negative as video stimuli, after the subjective evaluation from 800 short videos. The duration of each short video is about 1–3 min. In the experiment, the emotion types of two adjacent videos are different and the videos are played pseudo-randomly, and 2,495 samples had been collected for each subject. With the 14-channel EEG signals obtained, the band-pass filter is used for 0.10–50 Hz frequency filtering, and the notch filter is employed for denoising. And then the pre-processed EEG signals are cut into 1-s segments and the DE features are also extracted.

For the experimental paradigm on the self-collected dataset, after filling in basic information, the subject needs to read the experiment description and completes a 5-min baseline recording with opening or closing eyes alternately every 15 s. During a 15-s baseline recording, the subject is required to remain relaxed, blink as infrequently as possible, and look at a fixation cross “+” on the screen. Then a video stimulus is displayed and the subject is asked to stay as still as possible and blink as infrequently as possible when watching the stimulus. After that, the subject filled in the subjective evaluation scales based on his immediate true feelings. To eliminate the effect of the previous stimulus, the subject is asked to complete two simple calculation questions within ten as a distraction. Next, a more than 30-s period of rest is taken, during which a blank screen is displayed and the subject is asked to clear his brain of all thoughts, feelings and memories as much as possible. When the subject clicks the “NEXT” button on the screen, the next trial starts. The above process is repeated until 12 short videos have been played.

In the experiments, we use the leave-one-subject-out verification method on the SEED, SEED-IV, and self-collected datasets, and employ all sessions of the subjects in the source domain as the source data. For the three-category SEED and self-collected datasets, the first three sessions are taken from target domain as the labeled data. For the SEED-IV dataset with four categories, since two adjacent sessions may have the same emotion category, we use the first several sessions from target domain as the labeled data until all emotion categories have presented. In the target domain, the data amounts used for calibration and testing are 674 and 2,720 in SEED, 499 and 323 in SEED-IV, 330 and 2,165 in self-collected dataset.

**Figure 5:**
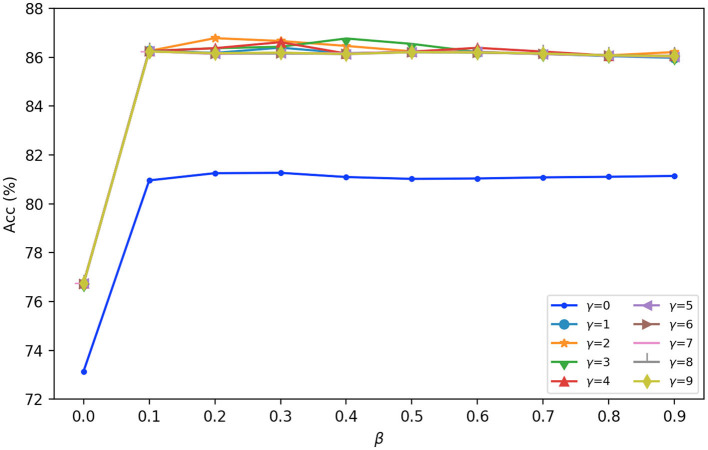
The accuracy results under different values of β and γ.

For the hyperparameters of β and γ in Equation (6), we set their values through the experiment. Take the SEED dataset as an example, Figure 5 presents the accuracy results under different values of β and γ. It can be seen from Figure 5 that, when γ = 0 and β = 0, the accuracy is only 73.14%; when γ = 0 and β>0, the accuracy is about 81%. When γ>0 and β>0, the accuracy fluctuates in a small range around 86%, and when γ = 2 and β = 0.2, the accuracy reaches the local maximum of 86.78%. The same to SEED-IV and self-collected datasets. Therefore, we set γ = 2 and β = 0.2 in the experiments. It is found that the fixed values of β and γ can be obtained to shorten the computing time without reducing the accuracy.

### 5.2. Offline experiments

#### 5.2.1. Analysis on the quantity selection of instances

**Figure 6:**
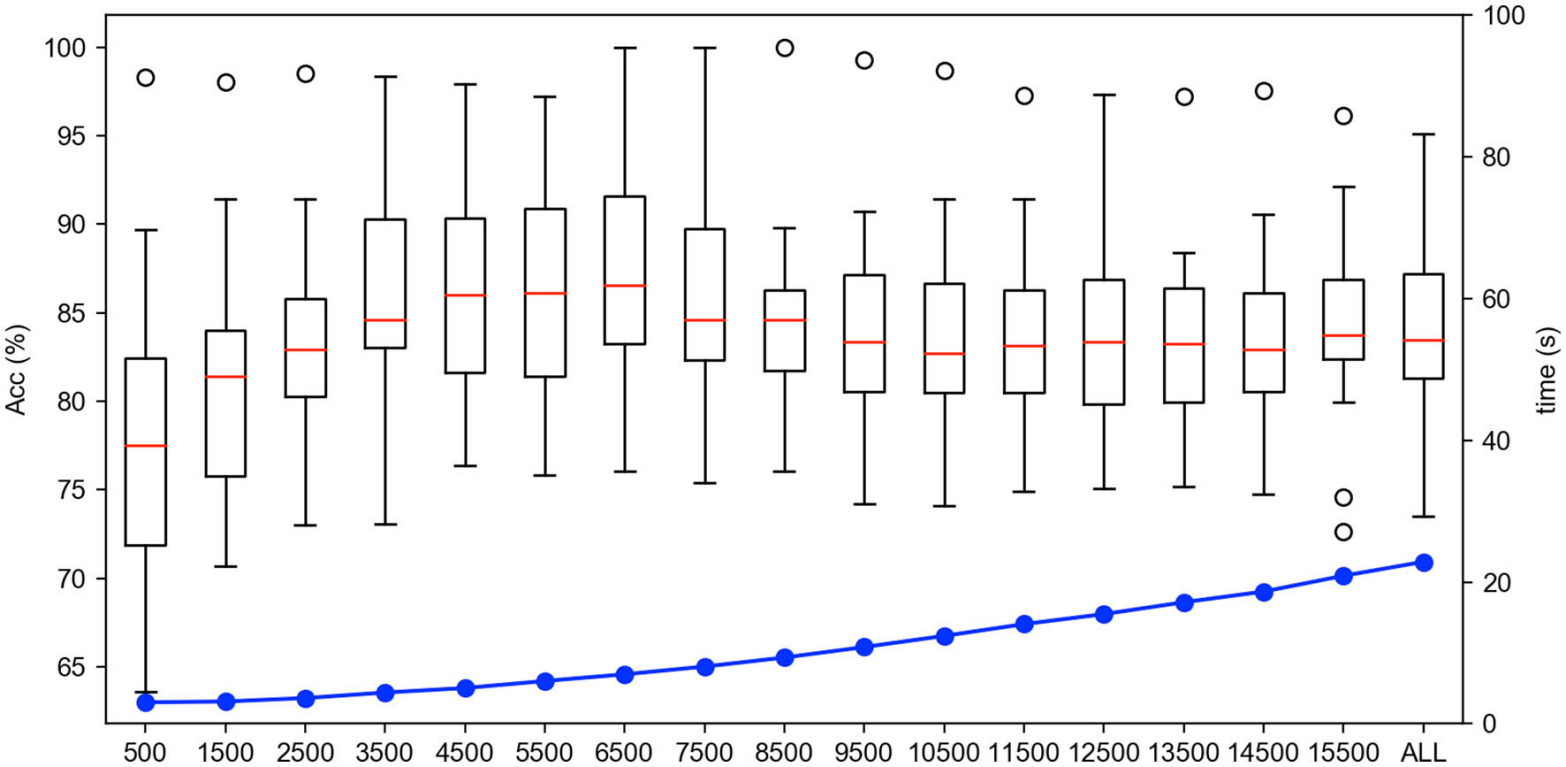
The accuracy and computing time of the proposed algorithm by selecting different number of instances on SEED dataset.

**Figure 7:**
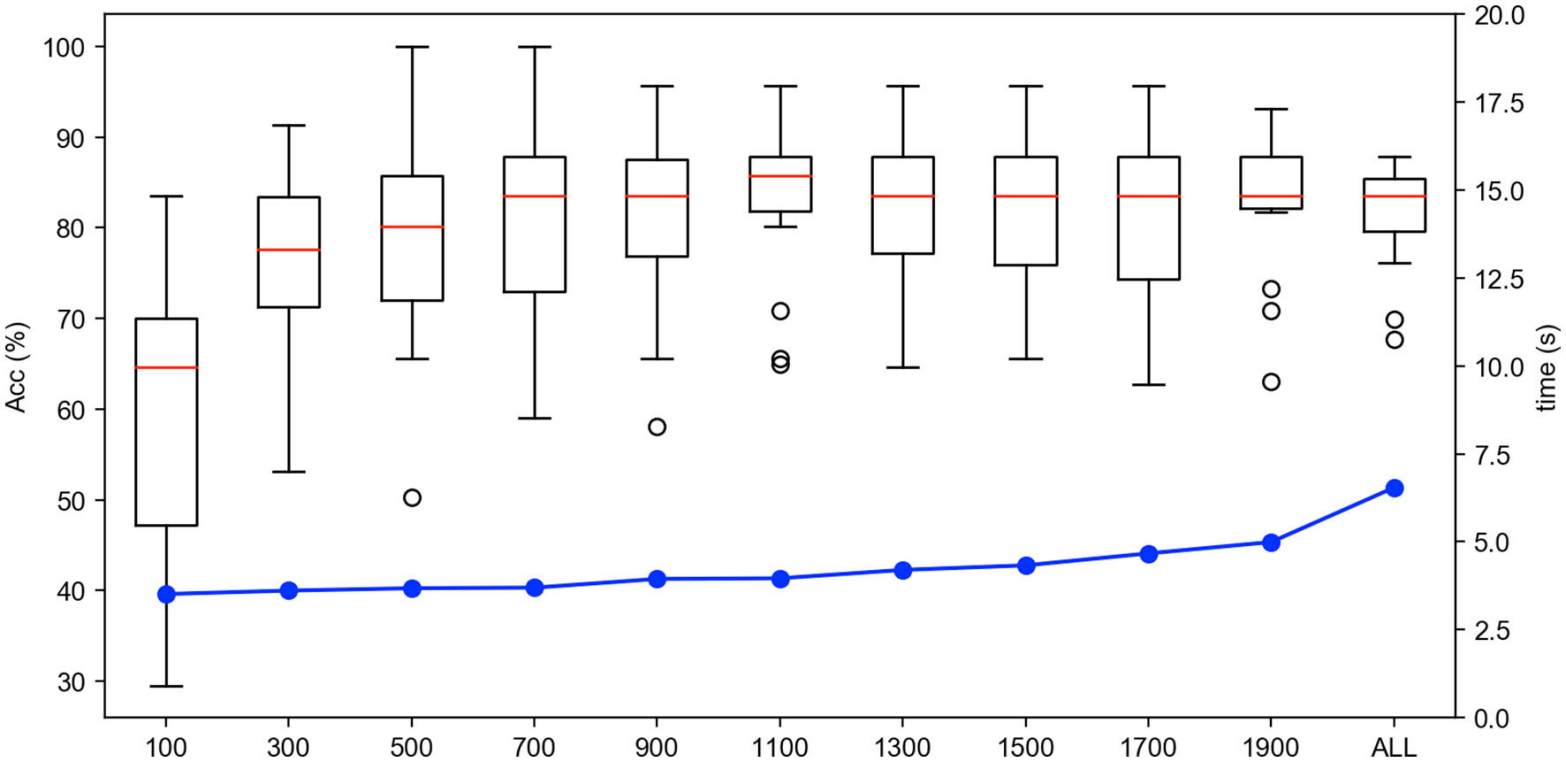
The accuracy and computing time of the proposed algorithm by selecting different number of instances on SEED-IV dataset.

**Figure 8:**
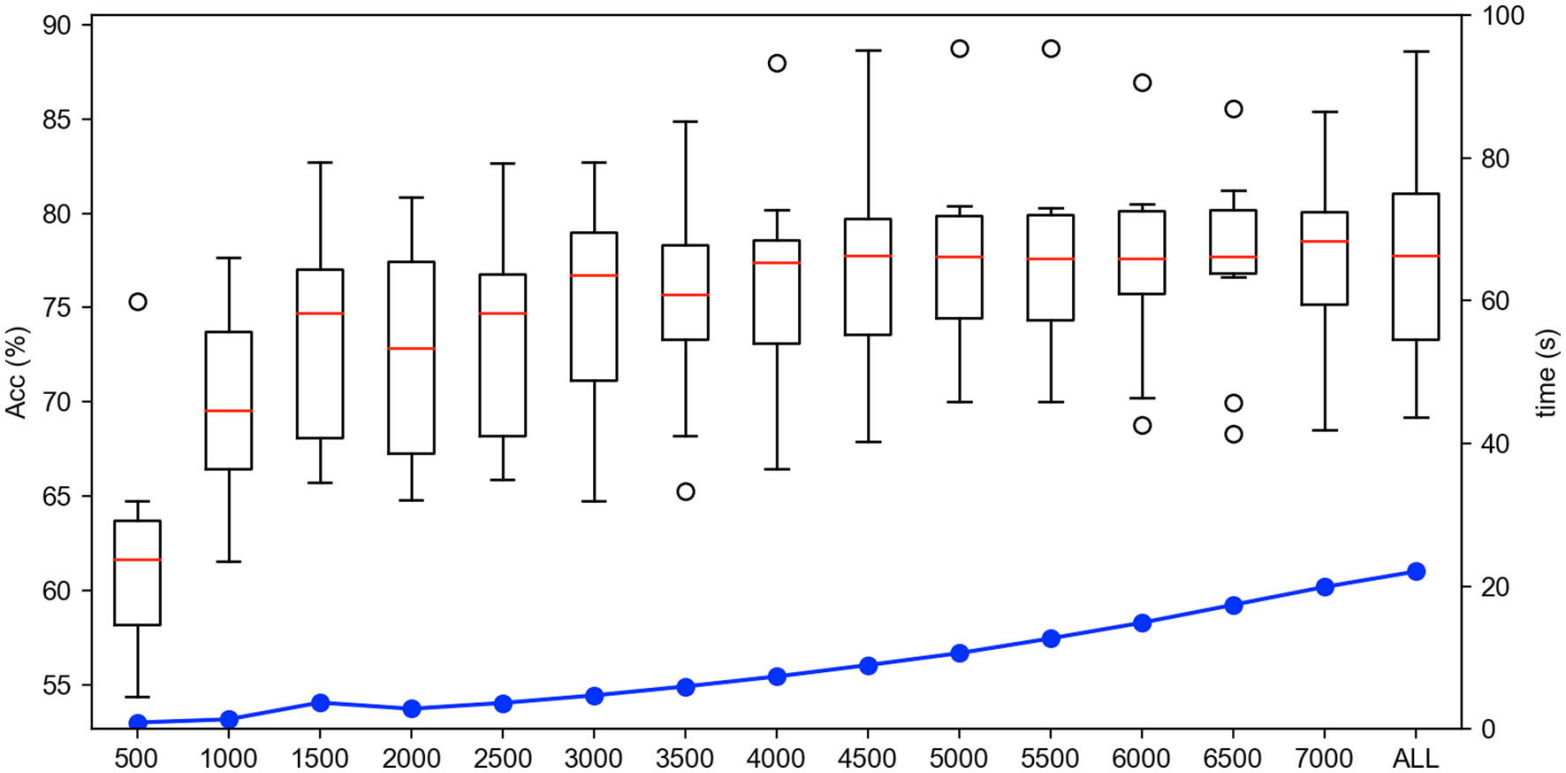
The accuracy and computing time of the proposed algorithm by selecting different number of instances on self-collected dataset.

Firstly, we test the effect of the number of instance selection on the performance of emotion recognition. Here, the incremental number of instances is selected according to the size of the dataset. Concretely, we select the number of instances from 500 to all with the incremental number being 1,000 for the SEED dateset, from 100 to all with the incremental number being 200 for the SEED-IV dataset, and from 500 to all with the incremental number being 500 for the self-collected dataset. The experimental results are shown in Figures 6–8, respectively.

In Figures 6–8, the horizontal axis corresponds to the number of selected instances *k* for each emotion category. The box denotes the accuracy under different values of *k* corresponding to the left vertical axis, and the blue line represents the computing time of model under different values of *k* corresponding to the right vertical axis. For the term of computing time, it is increased as the number of selected instances increases, as shown in the blue line. For the term of accuracy, it can be seen from Figures 6–8 that, it is not the more the number of instances is, the higher the recognition accuracy is. The accuracy can be rapidly improved before the number of selected instances reaches a certain value, which indicates that the selected instances during this period is informative and can be well-transferred to improve the generalization ability of the model. However, when the number of the instance exceeds this certain value, the improvement of the accuracy slows down until reaching the maximum value, tending to be flat and decrease. This indicates that too many instances selected will also cause data redundancy and lead to negative transfer, degrading the performance of the model. It can be seen from Figures 6–8 that on the SEED, SEED-IV, and the dataset collected by ourselves, the selection number *k* in Equation (3) is, respectively, chosen to be 6,500, 1,100, and 5,500 for the instances with different emotion categories to train the model, which can obtain higher accuracy and lower standard deviation with 86.78 ± 6.65%, 82.55 ± 8.48%, and 77.68 ± 5.13% in computing time of 7, 4, and 10 s, respectively.

#### 5.2.2. Ablation experiments

As illustrated in Figure 2, the key components of the proposed SSTM-IS are instance selection and SSTM. To evaluate the effects of these two components on the SSTM-IS, we remove one at a time and evaluate the performance of the ablated models. The experimental results of the proposed SSTM-IS and two ablated models are summarized in Table 1. Here, the model of SSTM indicates to use all the source domain data for transfer learning without the component of instance selection. And the model of instance-sel represents to use the selected instances to perform the classification directly without transfer learning. It can be seen from Table 1 that on the SEED, SEED-IV, and self-collected datasets, the proposed SSTM-IS achieves the best accuracies of 86.78, 82.55, and 77.68%, the model of SSTM obtains the accuracies of 84.02, 81.66, and 76.96%, and the model of instance-sel gets the accuracies of 79.66, 64.22, and 70.31%, respectively. This indicates by jointly using two components of SSTM and instance selection, the proposed SSTM-IS algorithm can achieve the best performance on the SEED, SEED-IV, and self-collected datasets. In addition, the component of SSTM has a stronger impact on the performance compared with the instance selection, verifying the effectiveness of transfer learning. Moreover, the impact of instance selection on SEED is greater than that on SEED-IV. This may be because the SEED has a large amount of data and more redundancy in source domain, and thus the instance selection is more effective than that on the SEED-IV.

**Table 1:** Performance comparisons with the existing typical EEG emotion recognition algorithms on SEED and SEED-IV datasets.

**Dataset**	**Method**	**Acc (%)**	**Runtime (s)**
SEED	TCA (Pan et al., 2010)	64.24 ± 15.34	298
	CORAL (Sun and Saenko, 2016)	63.59 ± 7.60	19
	MS-MDA (Chen et al., 2021)	85.04 ± 7.85	1,959
	PPDA_NC (Zhao et al., 2021)	85.40 ± 7.10	-
	PPDA (Zhao et al., 2021)	86.70 ± 7.10	-
	DResNet (Ma et al., 2019)	85.30 ± 8.00	-
	MS-STM (Li et al., 2020a)	83.22 ± 13.96	776
	ASFM (Nandi et al., 2021)	83.51 ± 10.18	-
	FOIT (Chen et al., 2022)	82.05 ± 12.36	32
	SSTM	84.02 ± 5.38	22
	Instance-sel	79.66 ± 8.15	**5**
	SSTM-IS	**86.78** **±** **6.65**	7
SEED_IV	TCA (Pan et al., 2010)	29.06 ± 2.16	155
	CORAL (Sun and Saenko, 2016)	32.79 ± 12.25	43
	MS-STM (Li et al., 2020a)	80.28 ± 9.93	833
	FOIT (Chen et al., 2022)	78.15 ± 7.31	20
	SSTM	81.66 ± 6.10	7
	Instance-sel	64.22 ± 17.80	**3**
	SSTM-IS	**82.55** **±** **8.48**	4
Self-collected	TCA (Pan et al., 2010)	47.04 ± 4.91	203
	CORAL (Sun and Saenko, 2016)	46.26 ± 2.56	31
	MS-MDA (Chen et al., 2021)	68.98 ± 4.91	347
	MS-STM (Li et al., 2020a)	67.83 ± 4.75	89
	SSTM	76.96 ± 4.08	9
	Instance-sel	70.31 ± 5.62	**6**
	SSTM-IS	**77.68** **±** **5.13**	10

The bold values indicate the best performance in the current dataset.

#### 5.2.3. Comparison with other methods

In order to prove the effectiveness of the proposed algorithm, we compare several representative algorithms with our method on the public datasets of SEED and SEED-IV as well as the dataset collected by ourselves. The experimental results are shown in Table 1 in terms of accuracy and runtime. Among these representative methods compared in Table 1, MS-MDA (Chen et al., 2021), PPDA (Zhao et al., 2021), and DResNet (Ma et al., 2019) are incorporated as the benchmark algorithms by using deep learning. It should be noted that in Table 1, the results of MS-MDA (Chen et al., 2021) are the reproductions with the open source codes on the SEED dataset. The accuracies and runtime of TCA (Pan et al., 2010), CORAL (Sun and Saenko, 2016), MS-STM (Li et al., 2020a), and FOIT (Chen et al., 2022) on SEED and SEED-IV are referenced from the results given in (Chen et al., 2022). In addition, the accuracies and runtime of TCA (Pan et al., 2010), CORAL (Sun and Saenko, 2016), MS-MDA (Chen et al., 2021), and MS-STM (Li et al., 2020a) on the self-collected dataset are the reproductions with their open source codes. All the experiments are implemented by using Python and a GPU of NVIDIA GeForce GTX 1080.

It can be seen from Table 1 that compared with the existing representative EEG emotion recognition algorithms, the proposed SSTM-IS algorithm can achieve an accuracy of 86.78 ± 6.65% on the SEED dataset, which is 0.09% higher than the current best performance PPDA algorithm (Zhao et al., 2021) with the standard deviation reduced by 6.34%. On the SEED-IV dataset, the accuracy of our method reaches 82.55 ± 8.48%, which is 2.83% higher than that of MS-STM (Li et al., 2020a) with the standard deviation reduced by 14.60%. On the self-collected dataset, the accuracy of our method reaches 77.68 ± 5.13%, which is 14.52% higher than that of MS-STM (Li et al., 2020a). On the other hand, the proposed method uses a small amount of target domain data to train the model for a new subject, and the runtime of model training is 7, 4, and 10 s on the SEED, SEED-IV, and self-collected datasets, respectively. Once the model training is completed, our algorithm only needs several milliseconds to recognize the emotion state of un-labeled EEG samples. However, the existing deep learning methods use back propagation as the optimization strategy and need a long time to train the model for a new subject. Specifically, it can also be seen from Table 1 that the runtime of the proposed SSTM-IS algorithm is much more less than that of the deep learning method of MS-MDA (Chen et al., 2021) on the SEED dataset (7 vs. 1959 s) as well as the self-collected dataset (10 vs. 347 s). This indicates that our method can quickly calibrate the model suitable for new subjects, greatly shortening the runtime and more suiting for the real-time EEG emotion recognition system.

#### 5.2.4. Additional evaluations

**Figure 9:**
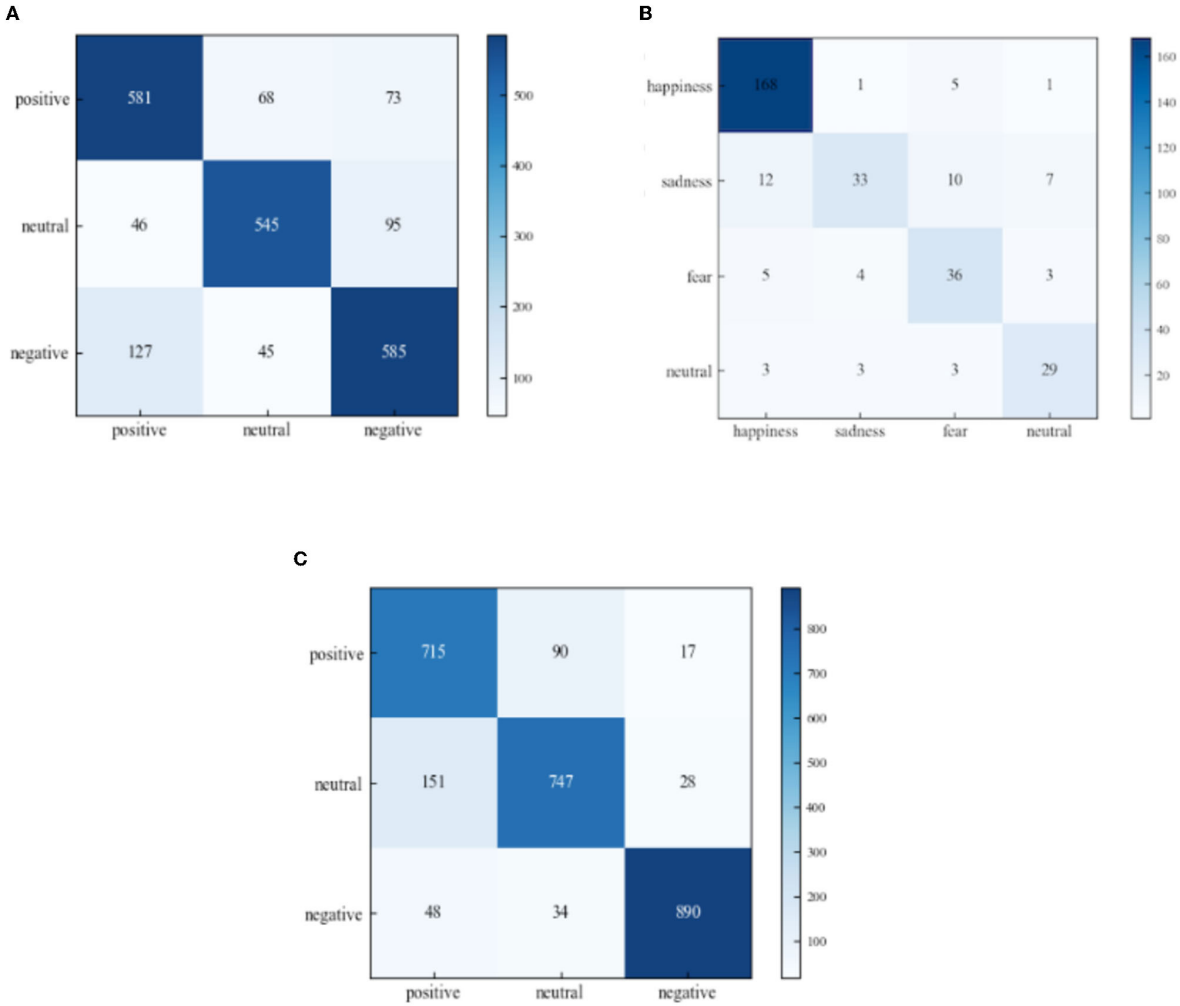
Confusion matrix of the proposed method in cross-subject on **(A)** SEED, **(B)** SEED-IV, and **(C)** self-collected datasets.

To analyze the recognition ability of the proposed algorithm for different emotion categories, the confusion matrix of predictions made on the SEED, SEED-IV, and self-collected datasets are shown in Figure 9. It can be seen from Figure 9A that on the SEED dataset, with the instance number being 6,500 for different emotion categories, the projection effects from source to target show difference between the different emotion recognition. Specifically, our method achieves the recognition accuracies of 78.22, 85.76, and 95.19% for the emotion categories of positive, neutral, and negative, respectively. This difference is also shown on the SEED-IV and self-collected datasets. For the SEED-IV dataset as shown in Figure 9B, our method can obtain the recognition accuracies of 89.36, 80.49, 66.67, and 72.50%, respectively for the emotion categories of happiness, sadness, fear and neutral. And for the self-collected dataset as shown in Figure 9C, our method can obtain the recognition accuracies of 77.06, 82.83, and 77.69%, respectively for the emotion categories of positive, neutral, and negative. The results on the SEED, SEED-IV, and self-collected datasets indicate that the proposed algorithm has strong discriminative capability for different emotion categories.

**Figure 10:**
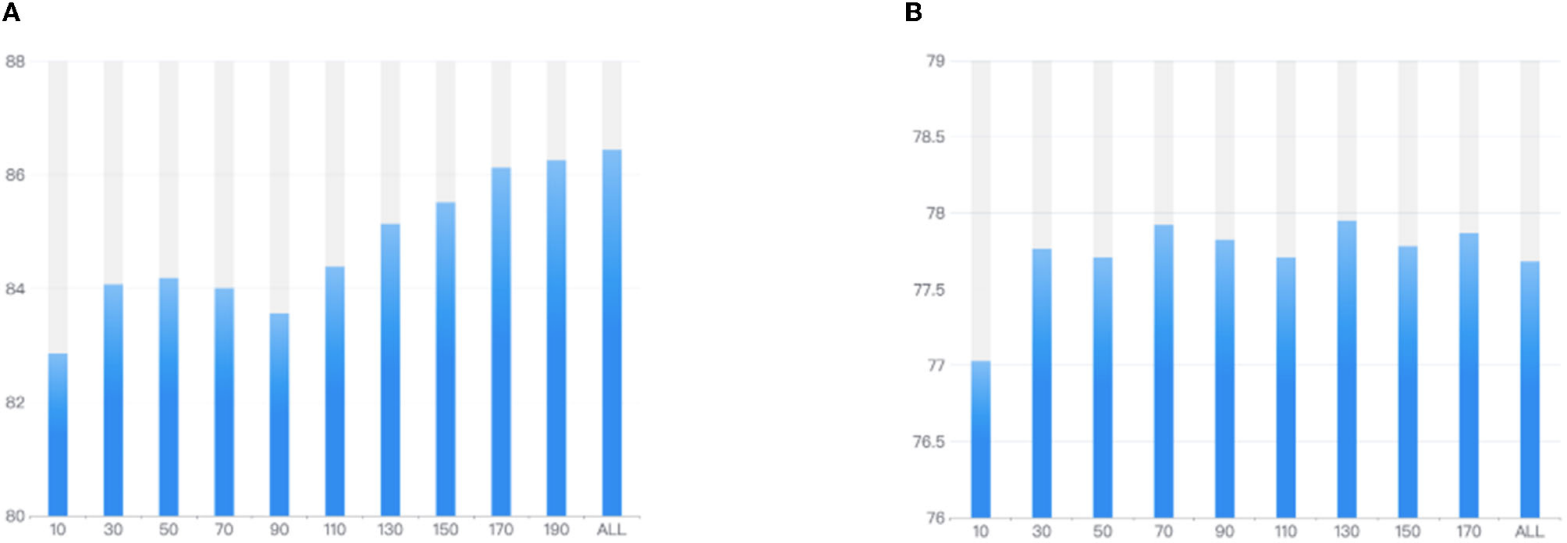
The recognition accuracies corresponding to different numbers of selected instances used to calibrate the model on **(A)** SEED and **(B)** self-collected datasets.

In addition, the proposed SSTM-IS algorithm needs a small amount of the labeled target domain data for supervised learning when training the model. In the previous experiments, we used the EEG data collected under the first three stimulus videos for the supervised learning. However, in the real-time emotion recognition applications, the less supervised data needed means the better experience for user. Therefore, we explore the impact of the number of selected instances used for supervised learning on the recognition accuracy by adding the instances collected within 20 s successively on the SEED and self collected datasets. The experimental results are shown in Figure 10. It can be seen from Figure 10 that, the recognition accuracy increases with the number of instances supervised until it tends to be flat and fluctuates in a small range. This gives us the insight, that is, a balance between the accuracy and user experience can be found in practice by selecting the number of instances where the accuracy levels off, reducing the amount of supervised data in the target domain to calibrate the model.

**Figure 11:**
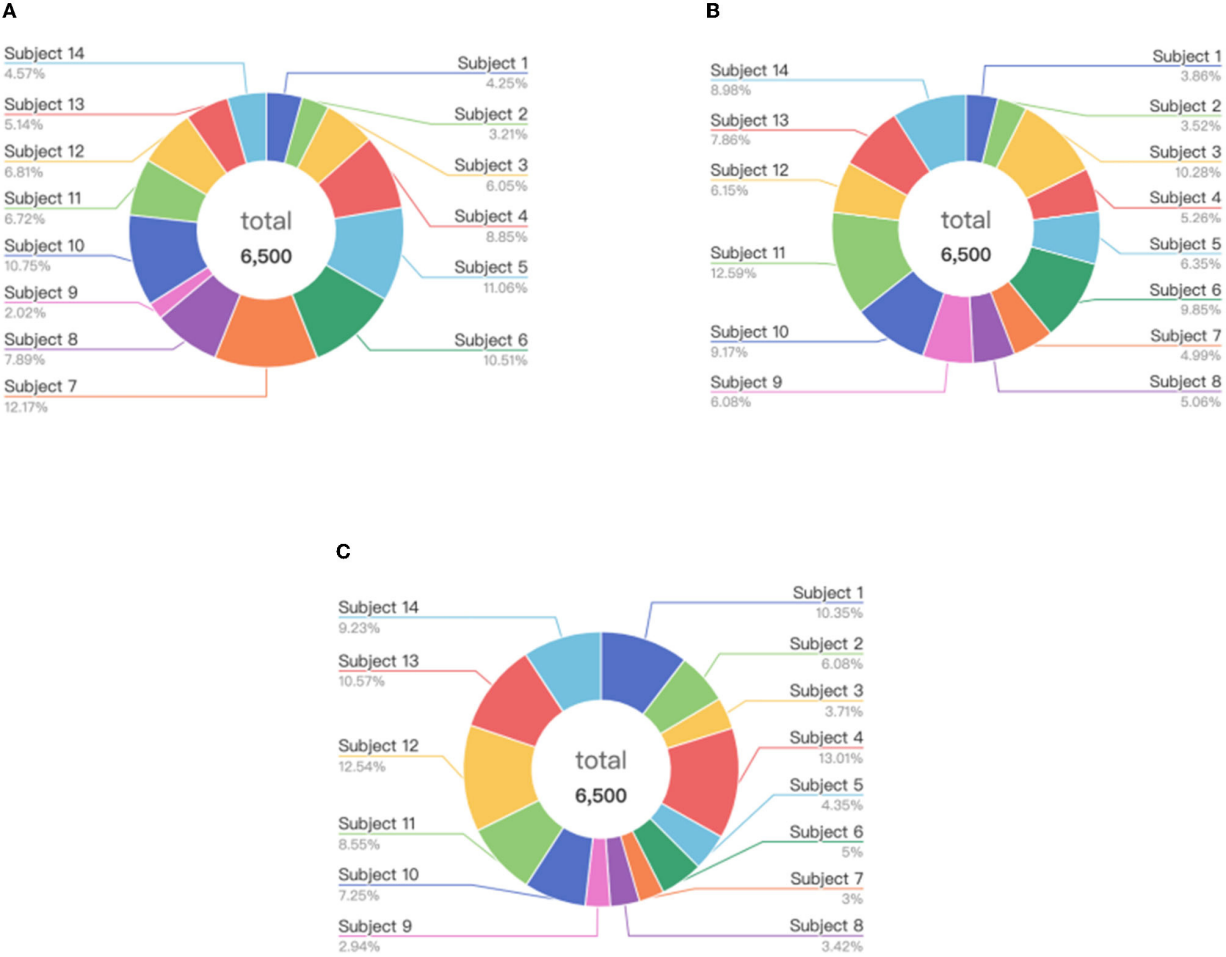
Distribution of the selected instances from the subjects on the SEED: **(A)** positive, **(B)** neutral, and **(C)** negative.

**Figure 12:**
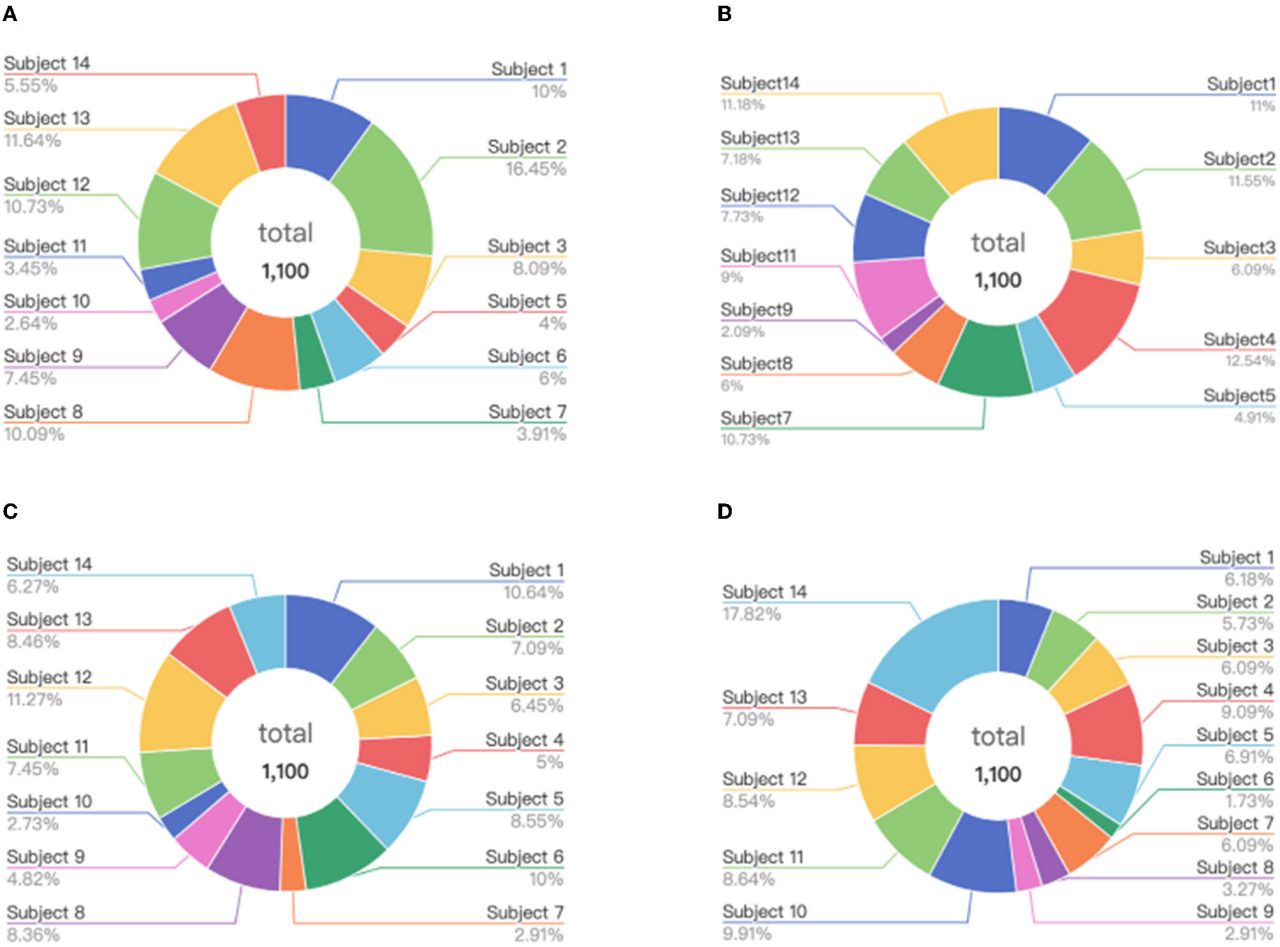
Distribution of the selected instances from the subjects on the SEED-IV: **(A)** happy, **(B)** sad, **(C)** neutral, and **(D)** fear.

**Figure 13:**
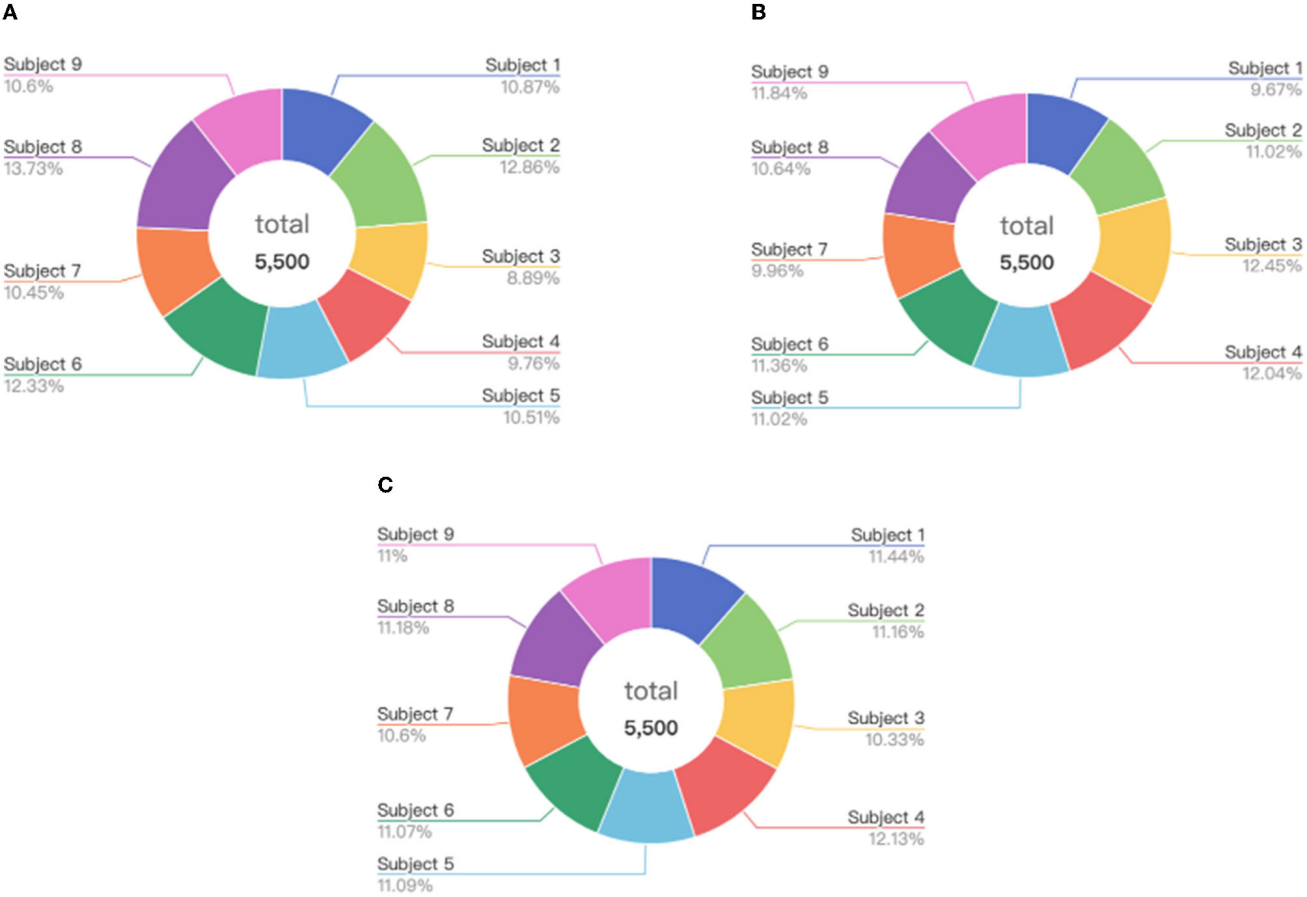
Distribution of the selected instances from the subjects on the self-collected dataset: **(A)** positive, **(B)** neutral, and **(C)** negative.

Considering the experimental results shown in Section 5.2.1, where the higher accuracy and lower deviation can be obtained for the selected instances of 6,500, 1,100, and 5,500 from the source domains of the SEED, SEED-IV, and self-collected datasets, respectively, here we further explore the data distributions of these selected instances in the source domain. The visualization results are presented in Figures 11–13, respectively.

We can find from Figures 11–13 that for each of the three datasets, the selected instances come from almost all the subjects of the source domain. With the number of source domain instances being the same for each emotion category, the numbers of selected instances vary from subject to subject in the source domain between the different emotion recognition. This demonstrates that the instances from subjects contribute differently to the construction of emotion recognition model on the target domain. The proposed strategy of instance selection is capable of obtaining the most informative samples from different subjects in the source domain for transfer learning on the target domain, which increases the generalizability of the model and reduces the negative transfer caused by data redundancy effectively.

### 5.3. Online experiments

In order to verify the reliability of our algorithm in the actual scenes, we use the real-time EEG emotion recognition system developed in Section 4 to conduct the real-time emotion recognition experiment for practical application. For the experimental paradigm in the online experiment, the experimental process is similar to that on the self-collected dataset. It should be noted that, the first three sessions of EEG data are collected from the new subject as the labeled target domain data to calibrate the model by the server. After the model calibrating is completed, the emotion recognition results will be displayed in real time for the experimenters.

**Figure 14:**
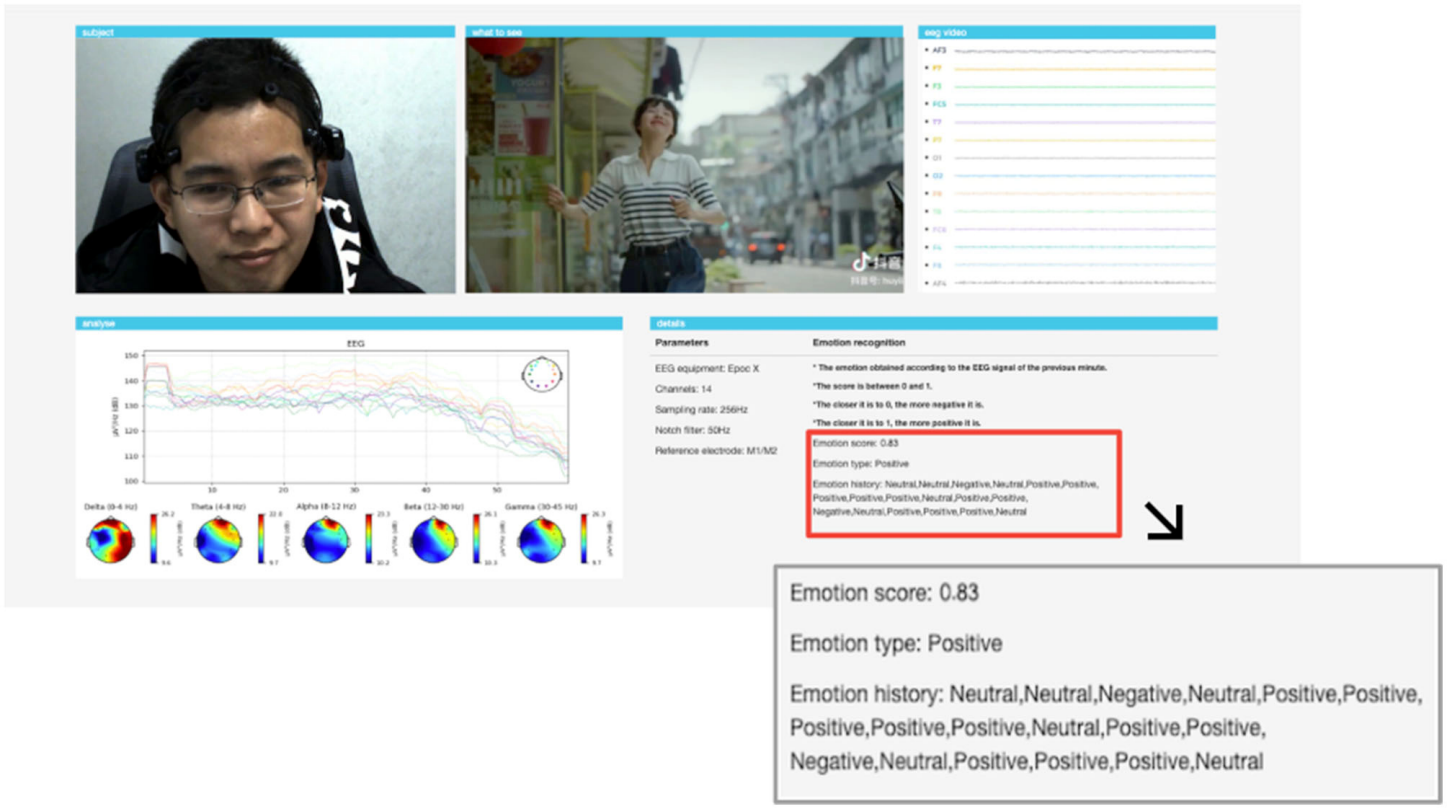
The visualization interface of online EEG emotion recognition system.

For the actual online situation as shown in Figure 3, we pre-process the raw data within every segment of 10 s and extract the DE features to put into the trained model for real-time emotion recognition. In the experiment, we divide the emotion states into three categories: positive, neutral, and negative. The visualization interface of the realistic experiment is shown in Figure 14. Here, the three windows in the first row are used for monitoring the subject state, stimulus display and real-time raw EEG signals. The window on the left of the second row shows the spectrum diagram and topographical maps of EEG signals, which are used to visualize the changes in frequency characteristics of EEG signals. And the window on the right shows the real-time and historical emotion recognition results. In the actual experiment, when the model training is completed, the proposed system outputs an emotion prediction for every 10s EEG data segment. The real-time system developed above implements the transfer learning algorithm by using a small amount of data for model calibrating, and it has also been verified in the real-time emotion recognition applications in real scene. In addition, it should be explained that we think it is inaccurate to take the label of the whole stimulating video as the ground truth for every 10 s data segment, and thus we did not conduct quantitative analysis on the online accuracy. It can be inferred that the emotion varies sometimes very fast and is effected by many factors such as the individual difference and induced effect of stimulating video, the online accuracy will be lower than the experimental results on the offline dataset.

## 6. Discussion and conclusion

The above experiments indicate that the proposed SSTM-IS algorithm brings a solid improvement in the accuracy and computing time for EEG emotion recognition. The strategy of instance selection is used to obtain the informative data samples from the source domain, which shortens the computing time for training the model. At the same time, the simplified STM method can further reduce the time cost without decreasing the accuracy. The offline experiments on the public and self-collected datasets show that the proposed SSTM-IS has improved the accuracy with less time cost compared with the representative methods. Specifically, the accuracies on the SEED, SEED-IV, and self-collected datasets have been improved to 86.78 ± 6.65%, 82.55 ± 8.48%, and 77.68 ± 5.13%, respectively, and the computing time has been shortened to 7, 4, and 10 s. In addition, we also develop a real-time EEG emotion recognition system to carry out the actual online experiments. The online experimental results demonstrate that, the designed system with the proposed SSTM-IS can provide a practically feasible solution for the actual applications of aBCIs.

There are also some tips for future works. First, the proposed algorithm still needs to collect a small amount of data from new individuals for model calibration, which reduces the user experience to a certain extent. In the future, the real-time emotion recognition algorithms can be explored without calibration. Second, the data of new subjects can be incorporated selectively into the source domain to optimize the data composition in realistic scenarios. Besides, the developed system also reserves the acquisition interfaces of other physiological signals to facilitate the subsequent integration of multiple physiological signals to further improve the system performance.

## Data availability statement

The raw data supporting the conclusions of this article will be made available by the authors, without undue reservation.

## Ethics statement

The studies involving human participants were reviewed and approved by Research Project Ethical Review Application, State Key Laboratory of Media Convergence and Communication, Communication University of China. The patients/participants provided their written informed consent to participate in this study. Written informed consent was obtained from the individual(s) for the publication of any potentially identifiable images or data included in this article.

## Author contributions

SR and WZ: conceptualization and validation. SR: methodology and writing—original draft preparation. SR and DD: software and formal analysis. LY: investigation and visualization. DD: data curation. WZ: writing—review and editing. QZ: funding acquisition and supervision. All authors have read and agreed to the published version of the manuscript.
